# Advancing the Health and Nursing Knowledge of Asian and Pacific Islander People Through the Asian/Pacific Island Nursing Journal

**DOI:** 10.2196/41486

**Published:** 2022-08-09

**Authors:** Hyochol Ahn

**Affiliations:** 1 College of Nursing Florida State University Tallahassee, FL United States

**Keywords:** Asian-Pacific, Pacific Islands, Asia, nursing, health care, health education, health disorders, diseases

As the editor-in-chief, I am happy to announce the launch of a new member of JMIR Publications’ open access family of journals, the *Asian/Pacific Island Nursing Journal* (APINJ). Created to fill the gap between nursing science and behavioral/social sciences, APINJ offers a forum for empirical, theoretical, and methodological issues related to Asian American and Pacific Islander ethnic and cultural values and beliefs as well as the biological and physiological phenomena that can affect nursing care. APINJ serves as a voice for nursing and other health care providers for research, education, and practice. APINJ is included in PubMed, PubMed Central, the Directory of Open Access Journals, and Scopus. As an open access journal, APINJ follows a continuous publication model, and articles are published as soon as they have been peer-reviewed and copyedited.

Nursing in Asia and the Pacific Islands comprises a rapidly growing group of professionals, and the region represents the fastest growing minority group in the United States. According to the 2020 United States census [1], there are 20.6 million people who identify as Asian, Native Hawaiian, or Other Pacific Islander alone (not in combination with another race), making up 6.2% of the US population. The 2020 United States census shows that 19.9 million people identified as Asian alone and 4.1 million people identified as Asian in combination with another race; approximately 690,000 people identified as Native Hawaiian or Other Pacific Islander alone, but almost 900,000 identified as Native Hawaiian or Other Pacific Islander in combination with another race. Asian people in the United States include more than 20 distinct ethnic groups with different languages, cultures, customs, and histories. Despite these substantial numbers, information about these groups is sorely lacking in publications that examine their health disparities, immigration and acculturation challenges, health education needs, policy implications, and responses to varied interventions in acute care and community settings [2,3].

As the official journal of the Asian American / Pacific Islander Nurses Association, APINJ supports researchers, educators, and practitioners in addressing these critical information deficits by providing a quality, peer-reviewed, international forum for the exchange of knowledge in relation to Asian and Pacific Islander health and nursing care. APINJ features research papers; empirical, theoretical, and clinical articles; editorials; abstracts of recent dissertations; and conference summaries that relate to Asian American and Pacific Islander health and nursing written by those in the nursing and social sciences disciplines, such as clinical and developmental psychology, sociology, anthropology, social work, public health, education, genetics, pharmacology, infectious disease, oncology, cardiovascular disease, pulmonary function and disease, dermatology, wound healing, immunology, anesthesiology, endocrinology, gastroenterology, hematology, neonatology, nephrology, pathology, physiology, nutrition, pain management, sleep disturbances, dental health, and mental health.

Building on the JMIR foundation, the scope of APINJ includes, but is not limited to, methods, interventions, instrumentation, and educational techniques; theoretical foundations that increase the understanding of underlying mechanisms for changes in health and illness; biopsychosocial, spiritual, and ecological impacts on practice, education, and research; and policy issues as a result of rigorous research outcomes.

APINJ offers authors a rapid and thorough peer-review, professional copyediting, and professional production of PDF, XHTML, and XML proofs. This journal adheres to the same quality standards as our flagship journal, the *Journal of Medical Internet Research*.

## Abbreviations

APINJ: *Asian/Pacific Island Nursing Journal*
